# Intrathecal blood injection: a case report of a rare complication of an epidural blood patch

**DOI:** 10.1186/s12883-020-01763-8

**Published:** 2020-05-13

**Authors:** Joseph Seemiller, Sankeerth Challagundla, Travis Taylor, Ramin Zand

**Affiliations:** Department of Neurology, Neuroscience Institute, Geisinger Medical Center, 100 N. Academy Ave, Danville, PA 17822 USA

**Keywords:** Intrathecal injection, Epidural blood patch, Procedural complications, Low pressure headache

## Abstract

**Background:**

Intrathecal injection is a rare complication of spinal anesthesia and an underreported complication of epidural blood patches. Although there are other reported cases of intrathecal blood injection, these cases lack confirmatory imaging and others report injection of mixed blood with other agents.

**Case presentation:**

We present a case report of post-laminectomy cerebrospinal fluid leak who underwent epidural blood patch placement. CT and MRI brain imaging was obtained, depicting intrathecal blood products. The patient had subsequent seizures and respiratory distress, received supportive care, and returned to baseline after several days.

**Conclusion:**

The patient’s clinical course illustrates the potential complications of blood products within CSF, including seizures and respiratory distress, which improved with supportive care in this case. Importantly, to our knowledge, this is the only report that clearly depicts injection of purely blood products, without other confounding agents (such as gadolinium), into intrathecal space and with diffuse spread through the CSF as visualized on CT and MRI imaging.

## Background

The placement of an epidural blood patch is a common and generally well-tolerated procedure and complications are rare. Immediate post-procedural pain and subdural hematoma have been described in the literature [1, 2]. However, intrathecal blood injection is a very rare complication, with intrathecal dural puncture reported to occur in only 1–3% of patients during epidural anesthesia [3]. Here we present a case of symptomatic post-laminectomy cerebrospinal fluid (CSF) leak who underwent an epidural blood patch that resulted in convulsions and respiratory distress secondary to iatrogenic intrathecal blood injection.

## Case presentation

**Fig. 1:**
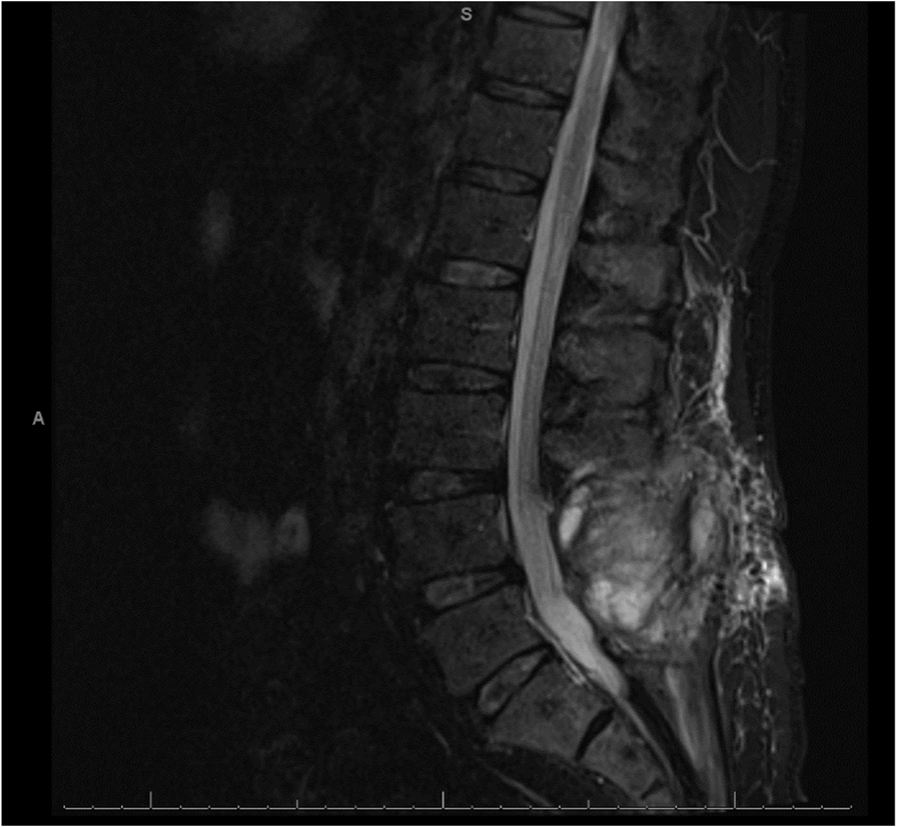
Non-contrast 1.5 T MRI STIR sequence of the lumbar spine, taken after surgery and prior to the blood patch procedure

A 56-year-old Caucasian man underwent an uncomplicated elective lumbar laminectomy for spinal stenosis, after which he developed a postural headache. Due to 2 months of ongoing post-laminectomy headache, a lumbar MRI was obtained that demonstrated increased extradural fluid around L4-L5 which did not clearly communicate with the thecal sac (Fig. 1). A CSF leak was suspected, and the patient was referred for an epidural blood patch. Prior to the blood patch, 3 mL of 1% lidocaine was injected for local anesthesia. The level of L5-S1 was found utilizing fluoroscopic guidance and 1 mL of Iohexol with intrathecal-like spread noted. Then, the blood patch was performed using 20 mL of autologous blood and a 20-gauge 3.5 in. Tuohy needle, utilizing loss-of-resistance technique.

Immediately post-procedure, the patient developed lower extremity spasms with refractory pain. The patient was then promptly referred to the nearby emergency department. Within 6 hours, he was unable to urinate, began repeating words, and he became verbally and physically abusive to staff and family. Nine hours after the procedure, his speech became increasingly dysarthric, which was followed by a generalized tonic-clonic seizure lasting 2 min. He subsequently developed respiratory distress, hypertension, and tachycardia, requiring mechanical intubation. He was also given intravenous levetiracetam at that time and the head of his bed remained elevated above 30 degrees.

**Fig. 2:**
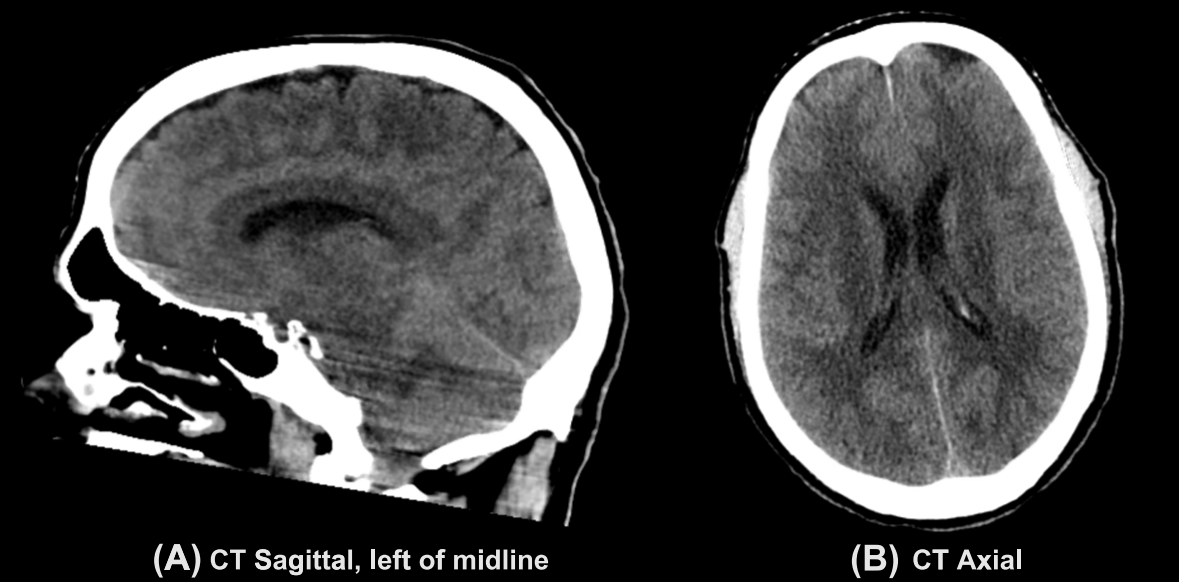
A CT head scan, taken upon the patient’s initial arrival to the emergency department, 12 h after intrathecal blood injection, in sagittal (**a**) and axial (**b**) planes. These images are remarkable for diffuse cortical sulci obscuration due to diluted blood products, with preserved grey white matter differentiation. The images can be wrongly interpreted as diffuse cortical edema

**Fig. 3:**
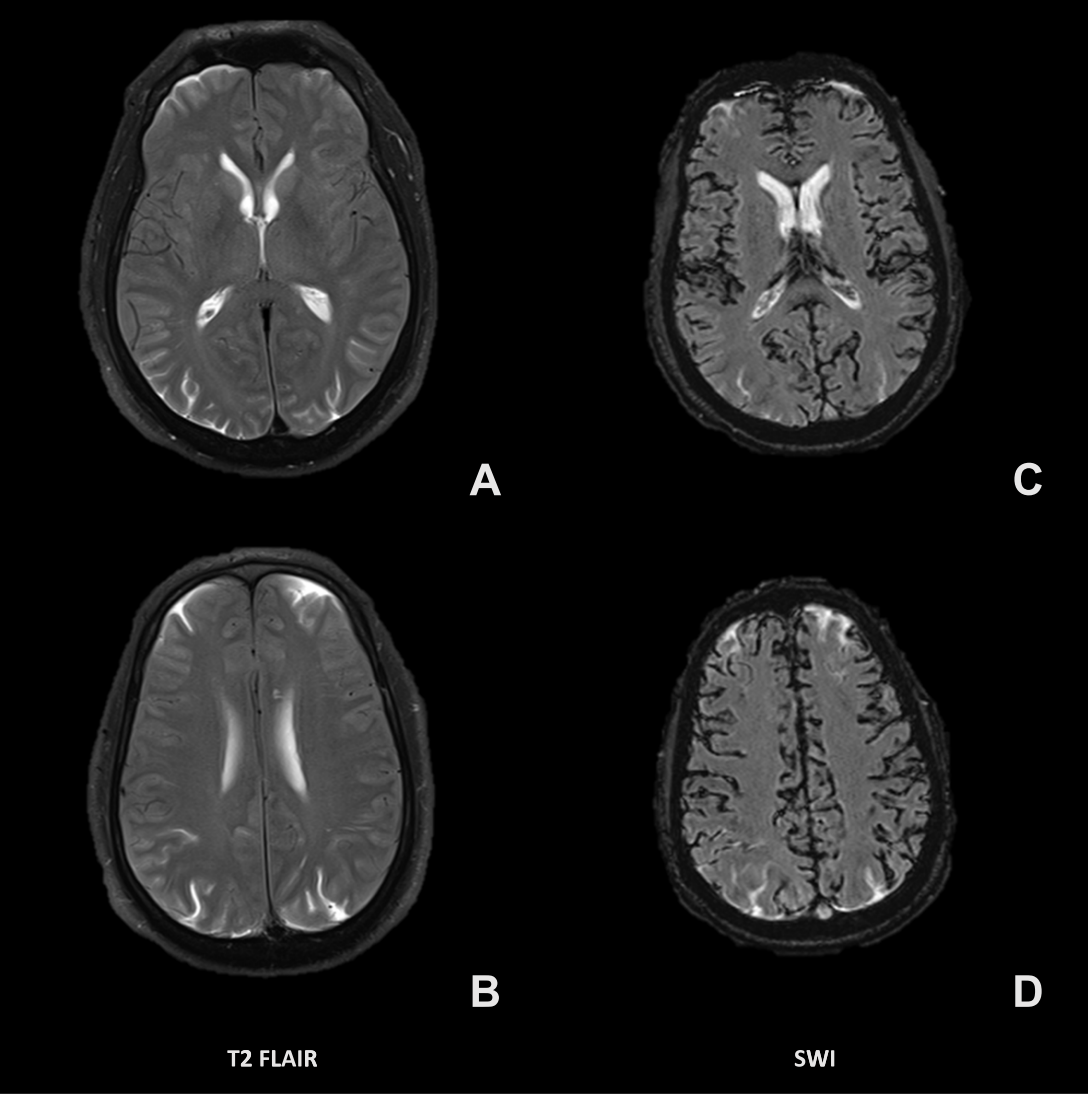
Non-contrast 1.5 T magnetic resonance imaging (MRI), taken 14 h after intrathecal blood injection, with both fluid-attenuated inversion recovery (FLAIR; **a**, **b**) and susceptibility weighted imaging (SWI; **c**, **d**)

Computed tomography (CT) of the head without contrast (Fig. 2) was obtained just under 12 h after blood injection. This imaging was remarkable for diffuse cortical sulci obscuration, with preserved grey white matter differentiation. Non-contrast 1.5 T magnetic resonance imaging (MRI) of the brain (Fig. 3) was obtained about 14 h after blood injection. On T2 fluid-attenuated inversion recovery (FLAIR), there was diffuse sulcal and ventricular hyperintensity consistent with diffuse spread of blood products or inadequately subtracted CSF. On susceptibility weighted imaging (SWI), there was diffuse susceptibility artifacts. This imaging was consistent with diffuse infiltration of blood products throughout the CSF. Additionally, MRI of the lumbar spine showed T1 hypointensity and T2 hyperintensity in the epidural and subarachnoid space, with T2 blooming artifact in the thecal sac. Lumbar spine imaging was consistent with blood products in both the epidural and subarachnoid spaces, possibly from mixed intrathecal and epidural blood injection, without hematoma.

The patient had an uneventful hospital course. EEG monitoring, initiated after his generalized tonic-clonic convulsions, revealed no further seizure activity. He was extubated on his third hospital day. He was discharged home on his fifth hospital day. At the time of discharge, he had no focal neurological deficits, but noted parasacral tenderness to palpation. At his three-month follow-up visit, he noted that his headache had completely resolved, and that he had no further seizures or other residual deficits.

The patient provided written informed consent for the publication of this case report.

## Discussion and conclusions

We present a case that illustrates complications of intrathecal blood injection, in which blood products were seen to spread diffusely through CSF. We detail the patient’s hospital course, involving progressive pain, agitation, and confusion, cumulating in a seizure and ultimately improving with supportive care. We also describe novel CT and MRI imaging of intrathecal injection of pure blood products, which has not been previously reported. Finally, we conducted a review of literature to better understand the clinical course and appearance of imaging after intrathecal blood injection.

This patient’s CT head imaging findings showed isodensity of blood products with obscured sulci. Based on this study alone, the differential diagnosis included hemispherical edema in addition to isodense subarachnoid hematoma (SAH). Subdural hematomas are typically isodense compared to brain parenchyma when they are chronic. However, there are rare reported cases of acute subdural hematomas that are isodense or hypodense to brain parenchyma, which are associated with anemia [4], disseminated intravascular coagulation, and dilution of blood products with CSF [5]. Our patient’s findings are most consistent with dilution of blood products with CSF given the clinical context of the preceding blood patch.

Inadvertent intrathecal injection is a rare complication of epidural blood patches. Blood patches are a standard of care for spinal headaches caused by needle-related dural puncture. However, they are more controversial when used for surgery-related dural leaks, although suggested to be safe in a case series [6]. Local complications include reactions caused by the mass effect of injected blood (radiculopathy, subdural or subarachnoid hematoma). Systemic reactions can result from entrance of a toxin into CSF and can include changes in level of consciousness and seizures.

One report described a blood patch given empirically due to headache starting directly after epidural bupivicaine given for childbirth [7]. This patient suffered from apnea but was able to be extubated in only 3 h after the blood patch and she rapidly became alert. CT demonstrated air collections in subarachnoid space, however, no MRI was reported. Although this presentation could have been related to spread of blood intrathecally, intrathecal migration of anesthetics cannot be excluded. In contrast, in our report, only 3 mL of 1% lidocaine was utilized for local anesthesia, and our imaging was consistent with diffuse infiltration of blood through the CSF.

A case report similar to our report describes a patient who received intrathecal gadolinium and an epidural blood patch, who subsequently had a grand-mal seizure and respiratory distress requiring mechanical ventilation. This patient’s hospital course was complicated by pneumonia, but she reportedly improved and was discharged on hospital day 10, with negative neurological exams on follow-up [8]. The paper’s authors attributed the neurological changes to gadolinium encephalopathy, however, there were also blood products seen on lumbar puncture, most likely secondary to the epidural blood patch. MRI brain showed diffuse intraventricular and intracerebral gadolinium, however, as the authors note, gadolinium and blood can have the same appearance. The effect of intrathecal blood products is unable to be excluded in this case report. Indeed, this patient’s hospital course, with acute-onset seizures and respiratory distress, improving with supportive care alone, strongly resembles our patient’s hospital course, in which gadolinium was not introduced into the CSF.

A further case report [9] of intrathecal gadolinium injection noted a similar hospital course, with disorientation, restlessness, aggressive behavior, and with visual and auditory hallucinations, without seizures and respiratory failure. MRI brain revealed diffuse enhancement in subarachnoid space. A lumbar puncture was performed; however, the presence or absence of CSF blood products was not mentioned. The patient improved with supportive care and was discharged 10 days after admission.

Multiple case reports [10–13] have detailed the outcome of subarachnoid injection of blood during spinal injections. Patients had fever, radicular pain, or lower extremity weakness. Outcomes ranged from complete resolution of symptoms in 10 days to chronic arachnoiditis and lower extremity weakness for multiple years. Lumbar imaging in these studies showed that the blood collection was limited to subarachnoid space, likely explaining the lack of severe mental status changes in these cases, in contrast to our case, in which mental status changes were likely secondary to the diffuse spread of blood products through CSF.

Although there are only a limited number of case reports similar to ours, the symptoms described in our case are consistent with those previously reported, particularly one report involving both intrathecal gadolinium and blood products [8] as noted above. However, in our report, gadolinium was not used, and therefore, our observed MRI changes were clearly secondary to blood products. This could suggest that certain acute mental status changes previously attributed to gadolinium could alternatively have been attributed to intrathecal blood products. Finally, we also present novel CT and MRI imaging that demonstrate the appearance of acutely injected intrathecal blood products, which, to our knowledge, has not previously been published.

The mechanisms of intrathecal blood causing mental status changes could overlap with those proposed for subarachnoid hemorrhage. In experimental rat models, a fluid with a composition similar to CSF was injected into the subarachnoid space of rats [14], leading to a cortical spreading depression and causing cortical ischemia, which was not seen in this patient, as well as microvascular vasospasm. Hemoglobin can also scavenge nitric oxide, reducing the levels of an important regulator of cerebral blood flow, resulting in vasospasm [15]. Although the mechanistic process is poorly understood, this supports the need for close observation of these patients, especially for complications of vasospasm, cortical ischemia, and seizures.

The mainstay of management for inadvertent intrathecal injections is cardiopulmonary supportive care. CSF lavage has been suggested as a therapy for intrathecal injection of ionic contrast agents, involving the removal of CSF and replacement with normal saline. Muscle relaxant and anticonvulsant therapy has also been proposed [16]. Maintaining the head of the bed in an upright position has been suggested in a case report of accidental ioxitalamate administration, to avoid cephalad migration of hyperbaric substances [17]. However it is not clear if the above strategies are helpful or even necessary in the case of intrathecal blood injection.

In this case, the patient’s head was maintained elevated above 30 degrees after he had a convulsive episode prompting mechanical ventilation. He was also started on levetiracetam after his convulsions, which was stopped at outpatient follow-up. It is unclear whether either of these interventions, or if earlier head elevation and levetiracetam therapy, would have lowered the risk of convulsions or impacted his hospital course. Additionally, the discussed mechanisms by which intrathecal blood could precipitate a change in clinical status could be dose-dependent, based on the volume of injected blood products. It is possible that a volume of blood lesser than 20 mL would have had less severe outcomes, however, there is no evidence to support that relationship.

Interventions for intrathecal injection management are untested in randomized control trials, and care should be taken when guiding care based on case reports. Due to the sporadic and emergent nature of these cases, it would be extremely difficult to conduct such a study. Therefore, some argue that considering these interventions on a cases-by-case basis is therefore prudent [18].

To our knowledge, this is the only report that clearly depicts injection of purely blood products, without other confounding agents (such as gadolinium), into intrathecal space with diffuse spread through the CSF. Further, this is the first report depicting the brain imaging of a rare complication of an epidural blood patch. Further descriptions of this rare complication are needed to fully understand its presentation and course. However, if back pain, radicular symptoms, changes in level of consciousness, or seizures develop after an epidural blood patch, subarachnoid or intrathecal injection should be suspected.

## Data Availability

Not applicable.

## Abbreviations

CSF: Cerebrospinal fluid
CT: Computed tomography
EEG: Electroencephalogram
FLAIR: Fluid-attenuated inversion recovery
MRI: Magnetic resonance imaging.
SWI: Susceptibility weighted imaging
